# *Eriophyes pouteriae* sp. nov., a New Mite Species Infesting *Pouteria sapota* †

**DOI:** 10.3390/insects15120972

**Published:** 2024-12-06

**Authors:** Marcello De Giosa, Enrico de Lillo, Aline D. Tassi, Alexandra M. Revynthi, Daniel J. de Andrade, Ronald Ochoa, Xiangbing Yang, Daniel Carrillo

**Affiliations:** 1 Tropical Research and Education Center, Department of Entomology and Nematology, University of Florida, Homestead, FL 33031, USA; m.degiosa@ufl.edu (M.D.G.); aline.tassi@ufl.edu (A.D.T.); arevynthi@ufl.edu (A.M.R.); 2 Department of Soil, Plant and Food Sciences (Di.S.S.P.A.), University of Bari Aldo Moro, 70121 Bari, Italy; enrico.delillo@uniba.it; 3 Departamento de Fitossanidade, Faculdade de Ciências Agrárias e Veterinárias, Universidade Estadual Paulista (FCAV/Unesp), Jaboticabal 14884-900, São Paulo, Brazil; daniel.andrade@unesp.br; 4 Systematic Entomology Laboratory, United States Department of Agriculture, Agricultural Research Service, Beltsville, MD 20705, USA; ron.ochoa@usda.gov; 5 Subtropical Horticulture Research Station, United States Department of Agriculture, Agricultural Research Service, Miami, FL 33158, USA; xiangbing.yang@usda.gov

**Keywords:** mamey sapote, bud mites Eriophyoidea, Eriophyidae, Eriophyinae, morphological description, molecular markers

## Abstract

*Pouteria sapota*, or “mamey sapote”, is a tropical fruit tree native to Central America and Southern Mexico, producing sweet, nutrient and vitamin-rich fruit. Several insect pests are known to infest *P. sapota,* but none have been associated with plant growth alterations. Eriophyoid mites are well known to cause plant malformations, but mites that cause this type of damage to mamey sapote have not been reported. Trees with abnormal leaf growth, including stunted leaves, outward curling, leaf yellowing, and diminishing overall tree vigor, were found in multiple locations in Southern Florida and one location in Brazil. Numerous plant samples were examined for the presence of minute eriophyoid-like mites, and a new species was found. It was morphologically described, and DNA fragments of the mitochondrial gene cytochrome c oxidase subunit I (COI), the nuclear subunit D2 region in 28S rDNA, and the ITS nuclear regions were PCR-amplified and sequenced. Morphological and molecular descriptions of the new species, named *E. pouteriae* sp. nov., are provided to aid the identification and future detection of this mite. Even though several species within the genus *Eriophyes* have been reported on other Sapotaceae species, this is the first eriophyoid mite known to be associated with mamey sapote.

## Simple Summary

Abnormal leaf growth, including stunted leaves, outward curling, leaf yellowing, and diminishing overall tree vigor of *Pouteria sapota* [(Jacq.) H.E. Moore et Stearn] (Sapotaceae) was observed in multiple locations in Southern Florida and one location in Brazil. In search for possible causal agents of these damages, a new mite species of the family Eriophyidae was found persistently infesting closed leaf buds of *P. sapota*. It was morphologically described and named *Eriophyes pouteriae* sp. nov.; DNA sequences of usual regions were also obtained. This is the first eriophyoid mite associated with *P. sapota*.

## 1. Introduction

*Pouteria sapota* [(Jacq.) H.E. Moore et Stearn] also known as “mamey sapote” or “mamey Colorado” is a tropical fruit tree belonging to the Sapotaceae family, putatively native to the lowlands of Central America and Southern Mexico [1]. It is an important fruit crop with commercial plantings in Miami-Dade, Florida (US), Mexico, Dominican Republic, Puerto Rico, and Cuba. Recently, small plantings have been established in other countries (e.g., Australia, Brazil, China, Israel, Philippines, Spain, Venezuela, and Vietnam). Cultivation is restricted to tropical and subtropical areas, as the plant is not tolerant to freezing temperatures. *Pouteria sapota* is appreciated and has long been cultivated as a dooryard tree throughout the tropical and subtropical Americas for producing a large and sweet, nutrient and vitamin-rich fruit [2], which was commonly named “chachaas” or “chachalhaas” by the Mayans and “tzapotl” by the Aztecs [3]. The entire mamey plant holds economic significance in various industries; its wood is used in furniture production, its leaves and stems provide chemical compounds for pharmacological and phytosanitary applications, its seeds are used in cosmetics, and its fruit pulp, both fresh and processed, serves as a food source [4,5,6]. Recently, the potential use of treated peel as a catalyst in the production of biodiesel has also been demonstrated, evaluated, and proposed [7].

Several insect pests are known to infest *P. sapota*, including the sapote fruit fly, *Anastrepha serpentina* (Wiedmann) (Diptera: Tephritidae), the Cuban may beetle, *Phyllophaga bruneri* Chapin (Coleoptera: Scarabeideae), and the sugarcane rootstalk borer *Diaprepes abbreviatus* L. (Coleoptera: Curculionidae), as well as several thrips species and scale insects [8]. Minor reports are communicated about spider mites (the Texas citrus mite *Eutetranychus banksi* (McGregor) and the two-spotted spider mite *Tetranychus urticae* Koch affecting this plant by leaf chlorosis [9,10], but no other mite taxa have been associated with *P. sapota*.

Abnormal leaf growth observed in commercial *P. sapota* plantings in Florida and Brazil was not associated with any macroscopically apparent agent, suggesting that very small mites could be involved. Eriophyoid mites are commonly overlooked because they are tiny and usually hide in different plant structures [11]. They frequently infest buds and young leaves, inducing plant malformations [12,13]. Their detection requires careful examination of samples, especially when present at low population density or inside architecturally intricate plant organs. Moreover, timely and reliable identification of Eriophyoid mites requires the application of specific protocols [14]. No eriophyoids are known from *P. sapota,* even though seven species of the genus *Eriophyes* have been associated with other species within the family Sapotaceae (Amrine & de Lillo, personal communication from unpublished databases). These include *E. emphlopei* Meyer (Smith) et Ueckermann and *E. nermae* Meyer (Smith) et Ueckermann, both found on *Sideroxylon inerme* L., *E. gallitor* Flechtmann et Etienne on *Sideroxylon obovatum* Lam., *E. hexandrae* Umapathy et Mohanasundaram and *E. manilkarae* (Keifer) on *Manilkara hexandra* Dubard, *E. mimusi* Meyer (Smith) et Ueckermann on *Mimusops zeyheri* Sond., and *E. planchonellus* Manson on *Pleioluma novocaledonica* (Dubard) Swenson et Munzinger. All these *Eriophyes* species induce the formation of closed and open galls, except for *E. planchonellus*, which causes distortions in flower buds [15,16,17,18,19].

This research aimed to discover and identify eriophyoid mites associated with *P. sapota* malformations and characterize them from a morphological and molecular point of view to facilitate future bio-ecological studies for their management.

## 2. Materials and Methods

### 2.1. Collection of Samples

**Figure 1:**
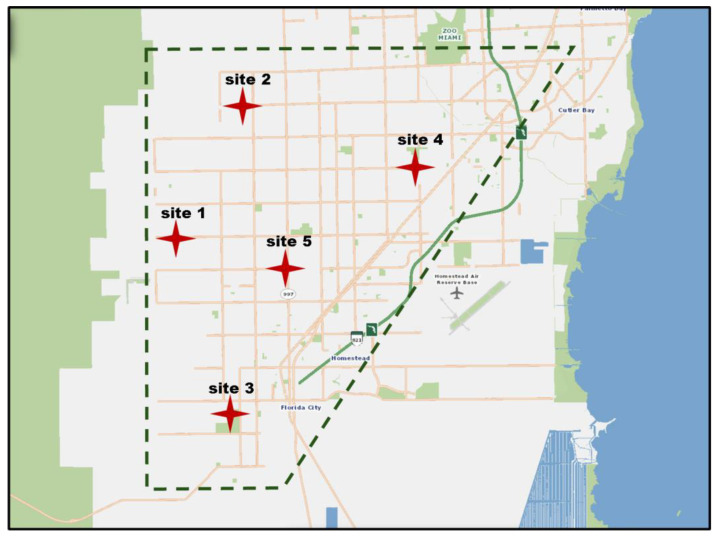
*Eriophyes pouteriae* sp. nov. found in five *Pouteria sapota* commercial orchards distributed throughout the Redland agricultural area in Homestead, FL. (site 1: 25°30′33.8976″, −80°30′25.2648″; site 2: 25°34′28.347″, −80°31′7.881″; site 3: 25°25′23.199″, −80°30′51.4008″; site 4: 25°33′53.0094″, −80°24′35.697″; site 5: 25°30′25.527″, −80°30′1.0332″).

Samples of *P. sapota* were collected during May–July, 2023, from 5 commercial orchards (ten samples per orchard) distributed throughout the Redland agricultural area in Homestead, FL, USA (Figure 1). Additional samples were obtained from *P. sapota* trees part of the germplasm bank of the São Paulo State University—Unesp, Campus of Jaboticabal, São Paulo State, Brazil, in September 2023. Similar samples from other plants in the family Sapotaceae, including *Chrysophyllum cainito* L. (caimito or star apple), and *Manilkara zapota* (L.)—P. Royen (sapodilla), were collected when available in the proximity of *P. sapota* trees.

### 2.2. Morphological Characterization by Light Microscopy

Samples composed of *P. sapota* leaves, stems, buds, and fruit were examined under a dissecting stereomicroscope (Nikon SMZ 1270, 400X, Nikon Instruments Inc., Melville, NY, USA) to determine the presence of mites, which were collected using a pin (Blick master synthetic round 3.0) and transferred to Eppendorf tubes containing Oudemans’ fluid [20]. Eriophyoid mites were cleared in Keifer’s booster and mounted in Keifer’s medium II [21]. The genus was determined using the generic key provided by Amrine et al. [22] and through comparisons with new genera described after that publication. The morphological terminology and setal notations followed Lindquist et al. [23]. The damage description followed that proposed by Keifer [24]. A phase contrast microscope (Olympus BX50, Tokyo, Japan) was used for taking line drawings and morphological measurements according to Amrine and Manson [25], as modified by de Lillo et al. [14]. Drawings and microphotographs were optimized using Adobe Photoshop© 10 version 21.0.3. Line drawings were made using a drawing tube as described in de Lillo et al. [14]. All measurements are given in micrometers (μm). For the adult female description, the holotype’s measurements are followed by the range values of the paratypes, which are reported in parentheses. Measurements are rounded off to the nearest integer and regard the length of the morphological traits unless otherwise specified. Abbreviations labeling line drawings follow Amrine et al. [22]. The host plant was identified by the Florida Department of Agriculture and Consumer Services Division of Plant Industry (FDACS-DPI), USA. The host plant name is in accordance with “The World Flora Online” [26].

Type materials are deposited at Acari reference collections of the Tropical Fruit Entomology laboratory (Tropical Research and Education Center, University of Florida), USA, FDACS-DPI, USA, the Smithsonian Natural Museum of Natural History, USA, and at the Department of Soil, Plant and Food Sciences (DiSSPA), University of Bari Aldo Moro, Italy (UNIBA). The holotype is deposited at UNIBA.

### 2.3. Morphological Characterization by Scanning Electron Microscopy

Leaves, stems, and buds of *P. sapota* were collected in October 2023 from a commercial orchard in Homestead, Florida, USA (25°30′33.8976″ N, −80°30′25.2648″ W). Mites were prepared and imaged with a TM 3030 Plus Tabletop Scanning Electron Microscope (Hitachi High Technologies America, Pleasanton, CA, USA) equipped with a Deben cooler system (Angstrom Scientific Inc., Ramsey, NJ, USA) following the methods of Otero-Colina [27]. Eriophyid specimens in situ on plant material were attached to 32 mm aluminum specimen holders with carbon sticky pads. Individual mites were removed from the plant material and mounted ventrally. The samples were cooled to −25 °C. An accelerating voltage of 5 KV was used to view the specimens. Micrographs were optimized using Adobe Photoshop© version 21.0.3.

### 2.4. Molecular Characterization—DNA Extraction, Amplification, and Sequencing

Genomic DNA was isolated individually from fifteen specimens collected from *P. sapota* using the DNeasy Blood Tissue Kit (Qiagen, Hilden, Germany). Live mites were dipped directly in 90 μL of ATL buffer (Qiagen); 10 μL of Proteinase K (Qiagen) was added to each sample and incubated at 56 °C and shaken in a thermomixer for 16 h. All other steps of the standard Qiagen DNA extraction protocol ‘Purification of Total DNA from Animal Tissues’ were modified according to [28]. Three target DNA fragments were PCR-amplified and sequenced per single mite: one mitochondrial gene, the cytochrome c oxidase subunit I (COI) [29], and two nuclear DNA fragments, the subunit D2 region in 28S rDNA [30,31] and the ITS nuclear region including IT1 and ITS2 (Table 1) [32,33]. 

PCR was conducted with 25 μL reaction volumes using a DreamTaq™ Hot Start polymerase system (DreamTaq™, Thermo Fisher Scientific, Waltham, MA, USA) following the manufacturer’s protocol. All PCR products were visualized on a 1.0% agarose gel stained with SYBR™ Safe DNA gel stain (Invitrogen, Waltham, MA, USA); in case of successful amplification, the remaining PCR products were run in a 1.5% low melting agarose gel stained with SYBR™ Safe DNA gel stain. DNA bands were excised with an x-trata™ gel extraction tool (Promega, Madison, WI, USA) and then purified using the Wizard^®^ SV Gel and PCR Clean-up System (Promega), according to manufacturer’s protocol. Both strand directions of the successfully amplified fragments (COI, partial 18S and 28S rRNA, and ITS) were Sanger sequenced (Eurofins Genomics, Louisville, KY, USA).

Staden Package v.1.6.0 [34] software was used to check the quality, and to edit and assemble the raw data into sequence contigs. Each marker’s nucleotide sequences were compared to those with the sequences of other *Eriophyes* available in the GenBank database using different blast algorithms (http://blast.ncbi.nlm.nih.gov/Blast.cgi accessed on 10 October 2024).

## 3. Results

### 3.1. Morphological Characterization

#### *Eriophyes pouteriae* sp. nov. (Figure 2) De Giosa, Tassi et de Lillo

*Description*

**Figure 3:**
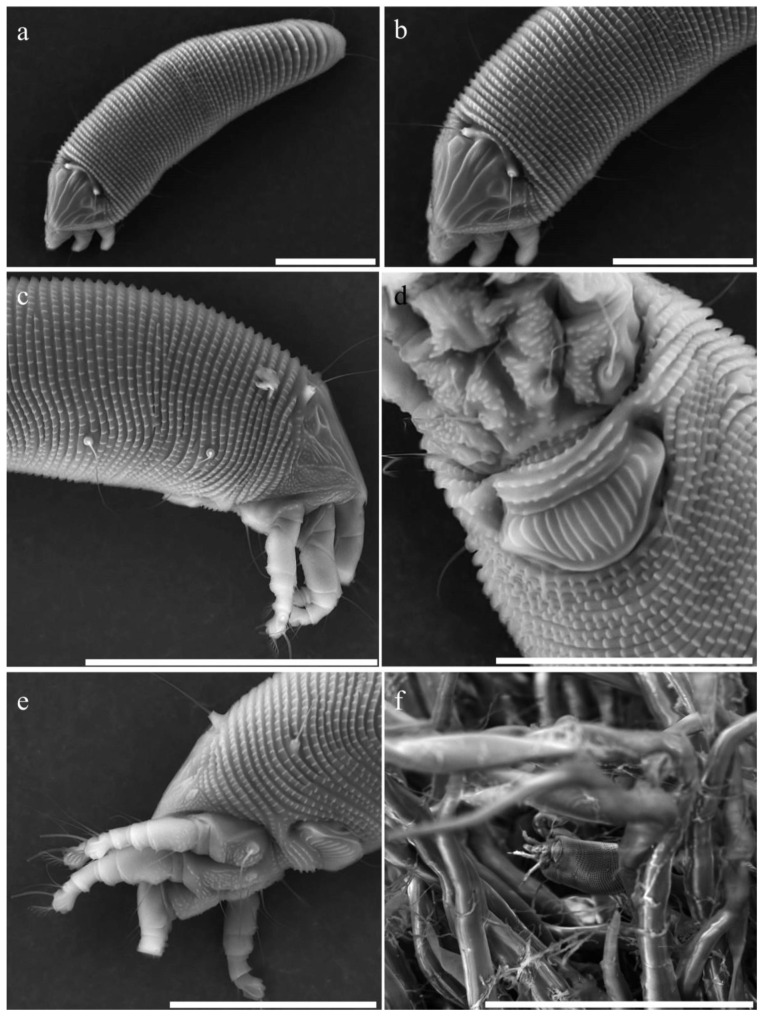
Scanning electron microscope images of adult female of *Eriophyes pouteriae* sp. nov.: (**a**,**b**) dorsal view, scales 50 µm and 30 µm, respectively; (**c**,**e**) lateral view, scales 50 µm and 30 µm, respectively; (**d**) coxigenital region, scale 20 µm; (**f**) female hiding within the trichomes of a plant bud, scale 200 µm.

FEMALE: (*n* = 11) Body vermiform (Figure 3a), 130 (122–164, from frontal lobe to anal lobes), 31 (28–44) wide, 40 (29–43) thick. Gnathosoma: palps 17 (17–20), palp coxal setae *ep* 1 (no range), dorsal palp genual setae *d* 4 (3–4), unbranched, palp tarsus setae *v* 1 (no range), cheliceral stylets 15 (14–16).

Prodorsal shield 24 (22–25, including frontal lobe), 22 (19–25) wide; with small rounded frontal lobe 1 (no range) over gnathosomal base. Prodorsal shield design with lines: interrupted median line on the anterior half and posterior quarter of the prodorsal shield; admedian lines complete; an inner pair of submedian lines complete and posteriorly ending with a loop, 3 shorter pairs of outer submedian lines; granules on the lateral sides (Figure 3b). Tubercles of scapular setae *sc* on the rear shield margin directed ahead, 10 (9–12) apart, scapular setae *sc* 22 (19–25) (Figure 3c). Leg I 22 (21–24, from base of trochanter), femur 9 (8–9), genu 4 (no range), tibia 5 (4–5), tarsus 5 (4–5), solenidion *ω* 6 (5–6, tapering), empodium 4 (no range), simple, 6-rayed; femoral setae 6 (6–7), genual setae *l″* 14 (13–16) and stiff, tibial setae *l′* 6 (4–6), tarsal setae *ft′* 12 (11–13), setae *ft″* 14 (13–16). Leg II 19 (17–21), femur 8 (7–8), genu 3 (3–4), tibia 3 (no range), tarsus 4 (4–5), solenidion *ω* 7 (6–7, tapering), empodium 4 (no range), simple, 6-rayed, femoral setae *bv* 6 (5–7), genual setae *l″* 8 (6–8), tarsal setae *ft′* 4 (3–4), setae *ft″* 17 (13–17). Coxae ornamented with several fine granules; setae *1b* 7 (6–9), tubercles *1b* 4 (3–5) apart, setae *1a* 15 (12–18), tubercles *1a* 5 (4–5) apart, setae *2a* 30 (25–32), tubercles *2a* 14 (12–15) apart. Prosternal apodeme 7 (6–7). Opisthosoma with 58 (53–62) dorsal semiannuli and 62 (58–65) ventral semiannuli; 6 (5–7) semiannuli with fine microtubercles between coxae and genital coverflap. Setae *c2* 12 (11–16), on ventral semiannulus 8 (7–8); setae *d* 26 (19–29), on ventral semiannulus 21 (21–24); setae *e* 22 (18–27), on ventral semiannulus 13 (11–55); setae *f* 13 (11–15), on ventral semiannulus 58 (55–64), 4 (no range) annuli after setae *f*. Setae *h2* 39 (36–45), setae *h1* 2 (1–2). Genital coverflap 9 (8–10), 17 (15–19) wide, coverflap with 15 (14–16) longitudinal striae, setae *3a* 7 (6–9), 11 (10–12) apart (Figure 3d,e). Internal genitalia: spermatheca quite elliptical, oriented postero-laterad; spermathecal tubes short as long as the spermathecal length; transverse genital apodeme trapezoidal and distally folded.

MALE (*n* = 1). Body vermiform, 102, 26 wide, 36 thick. Gnathosoma: palps 18, chelicerae 14. Prodorsal shield 20, including frontal lobe, 18 wide, frontal lobe 1. Shield pattern similar to that of female. Tubercles of scapular setae *sc* on the rear shield margin directed ahead, 8 apart, scapular setae *sc* 17. Leg I 20, femur 8, genu 3, tibia 5, tarsus 5, solenidion *ω* 6, tapering, empodium 4, simple, 6-rayed; femoral setae *bv* 5, genual setae *l″* 7, tibial setae *l′* 3, tarsal setae *ft′*, setae *ft″* 14. Leg II 19 (from base of trochanter), femur 8, genu 3, tibia 3, tarsus 4, femoral setae *bv* 3, genual setae *l″* 5, tarsal setae *ft′* 3, setae *ft″* 12, solenidion *ω* 7, tapering, empodium 4, simple, 6-rayed. **Coxae** similar to those of female; setae *1b* 5, tubercles *1b* 4 apart, setae *1a* 9, tubercles *1a* 4 apart, setae *2a* 17, tubercles *2a* 11 apart. Prosternal apodeme 7. Opisthosoma with 54 dorsal semiannuli; 63 ventral semiannuli; 5 semiannuli between coxae and genital region. Setae *c2* 9 on ventral semiannulus 8, setae *d* 19 on ventral semiannulus 18; setae *e* 13 on ventral semiannulus 31; setae *f* 31 on ventral semiannulus 9, 4 annuli after setae *f*. Setae *h2* 32; setae *h1* 2; setae *3a* 7, 8 apart.

*Type host plant—Pouteria sapota* (Jacq.) H.E. Moore et Stearn (Sapotaceae), mammey zapote.

**Figure 4:**
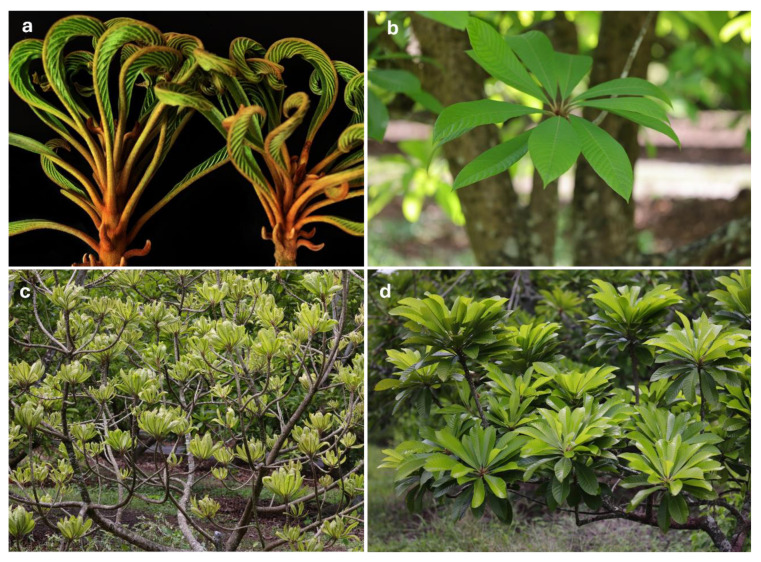
*Pouteria sapota* infested by *Eriophyes pouteriae* sp. nov. (**a**,**c**) and healthy (**b**,**d**): (**a**,**b**) detail of new leaves; (**c**,**d**) detail of branches of the tree.

*Relation to the host plant—Eriophyes pouteriae* sp. nov. was found inhabiting closed leaf buds, living between the vegetative apical buds (Figure 3f) in all *P. sapota* samples collected from different locations in Florida and Brazil. Infestations resulted in stunted leaves, leaves curling outward (clawing), and leaf yellowing (Figure 4a). Heavy infestations involve mite presence and damage to all new leaves on the entire tree and diminished tree vigor (Figure 4c). No mites were found on stems or fruit of *P. sapota* nor in any plant structure of other cultivated Sapotaceae, including *C. caimito* and *M. zapota*.

*Type locality—*Homestead, Florida, USA, 25°30′33.8976″ S, −80°30′25.2648″ W; 21 July 2023, coll. Carrillo Daniel and De Giosa Marcello.

*Type material—*Holotype: female on a microscope slide mounting a single specimen (indicated by an arrow on slide MS2306); paratypes: 25 females, 4 males, and 2 nymphs on 12 slides (slide codes from MS2301 to MS2312).

*Further materials—*Further specimens collected on the same location of the types are preserved in Oudemans’ fluid [19].

*Etymology—*The specific epithet, *pouteriae*, is based on the host plant species in the genitive case.

*Remarks—*Slide mounted specimens collected in Brazil are morphologically consistent with those described in Florida.

### 3.2. Molecular Characterization

Ten extractions yielded sequences of high quality for all three marker genes (COI DNA barcoding, ITS, and 18S and 28S rRNA partial sequence) of *E. pouteriae* sp. nov. Each sequence pair was found to be identical within their respective markers. Blastx search for COI sequences accs. numbers PQ167827, PQ182573-5 against Eriophyoidea GenBank returned as the best hit the sequence KY418168.1 (*Aceria* sp., 79.6% identity, 99% coverage); when sorted by identity, the best return hit was AKP20840.1 (*Eriophyes padi* (Nalepa), 83.15% identity, 87% coverage; host *Prunus padus* L. from Poland). For the marker ITS (PQ428922 PQ428924 and PQ428925), the best hit was OR139566.1 (*Aceria* sp., 96.92% identity, 19% coverage; host *Tamarix usneoides* E. Mey. ex Bunge from South Africa); and for the fragment including the partial sequence of 18S and 28S rRNA (accs. number PQ433866), the best hit was MK440017.1 (*Aceria lespedezae* Kuang, 89.63% identity, 78% coverage).

## 4. Discussion and Conclusions

*Eriophyes pouteriae* sp. nov. is the first eriophyid species associated with *P. sapota*. Seventeen Eriophyoid species have been described on plant species of the Sapotaceae family, including seven *Eriophyes* spp., and none of them have been reported in the Nearctic geographical area (Amrine & de Lillo, personal communication from unpublished databases). *Eriophyes pouteriae* sp. nov. is not morphologically similar to any of them. The closest species is *E. planchonellus* Mason, from which it differs in (1) the prodorsal shield size (as wide as long in *E. pouteriae* sp. nov. and one and a half times wider than long in *E. planchonellus*); (2) the prodorsal design (*E. pouteriae* sp. nov. has an incomplete median line and an inner submedian line posteriorly ending with a loop, whereas *E. planchonellus* is provided with a complete median line and an inner simple submedian line); (3) the coal ornamentation (sparse granules in *E. pouteriae* sp. nov. and very few dashes on the coxae II of *E. planchonellus*); (4) the number of semiannuli between the coxae and the external female genitalia (five–seven in *E. pouteriae* sp. nov. and four in *E. planchonellus*); and (5) the length of the opisthosoma setae (*c2*: 10–15; *d*: 19–29; *e*: 18–27; *f*: 12–15 in *E. pouteriae* sp. nov. and *c2*: 17–22; *d*: 30–37; *e*: 2–4; *f*: 15–19 in *E. planchonellus*).

*Eriophyes pouteriae* sp. nov. has a widespread distribution in Florida and is present in Brazil, suggesting that is probably present in other tropical and subtropical areas where *P. sapota* is cultivated, including its native range in Central America and Mexico. The finding of this mite inhabiting closed leaf buds of *P. sapota* and its absence on phylogenetically related plant species in the same area suggest that *E. pouteriae* sp. nov. specializes in feeding upon this plant. This high degree of specialization with a dominant monophagy is common in eriophyoid species [35]. However, additional sampling and host choice tests are required to fully understand the feeding habits, host range, and possible symbiotic associations of *E. pouteriae* sp. nov. The observed damage and abnormal leaf growth suggest that infestations by *E. pouteriae* sp. nov. can reduce the photosynthetic capacity of *P. sapota*. This is the only described species, along with *E. planchonellus*, that does not induce gall formation (galls), unlike the other *Eriophyes* species reported on Sapotaceae [24].

Public nucleotide sequences databases possess limited deposit data on *Eriophyes* species, including COI data of seven species, not necessarily linked to vouchers. Regarding ITS sequence only, no information is available, and for the 18S and 28S rRNA sequence fragments, just three representatives of the genus possess deposited nucleotide sequences. Blasting the obtained COI marker sequences against *Eriophyes* from GenBank showed the following information: for KT070227.1 *E. padi* (87% coverage and 80.72% identity), for MZ482942.1 *E. armandis* Xue et Hong (96% coverage and 76.82% identity), for MW691980.1 *E. calycobius* (Nalepa) (95% coverage and 74.92% identity), for *E. tiliae* (Pagenstecher) (85% coverage and 67.20% identity), for KU315235.1 *E. lissonotus* (Nalepa) (87% coverage and 72.58% identity), for EU254715.1 *E. pyri* (Pagenstecher) (69% coverage and 74% identity), and ON586803.1 *E. sorbi* (Canestrini) (84% coverage and 75.66% identity). Regarding the rRNA sequences, three species are available: for ON555745.1 *E. tiliae* (75% coverage and 90.86% identity), for MZ279784.1 *E. hoheriae* Lamb (69% coverage and 88.92% identity), and for ON555746.1 *E. sorbi* (71% coverage and 89.64% identity). Given the limited number of sequences deposited in GenBank for this genus within the Eriophyidae family, using BLAST tools for species identification and conducting phylogenetic studies are not feasible. Therefore, it is crucial to generate more data for further analysis. The information gathered on *E. pouteriae* sp. nov. in this study will contribute to enhancing the dataset for this genus, facilitating future research efforts.

Furthermore, adverse effects of *E. pouteriae* sp. nov. infestations on fruit production and yield have not been observed but warrant further investigation. In addition, sustainable management options should be explored to mitigate the damage caused by this mite.

## Figures and Tables

**Figure 2:**
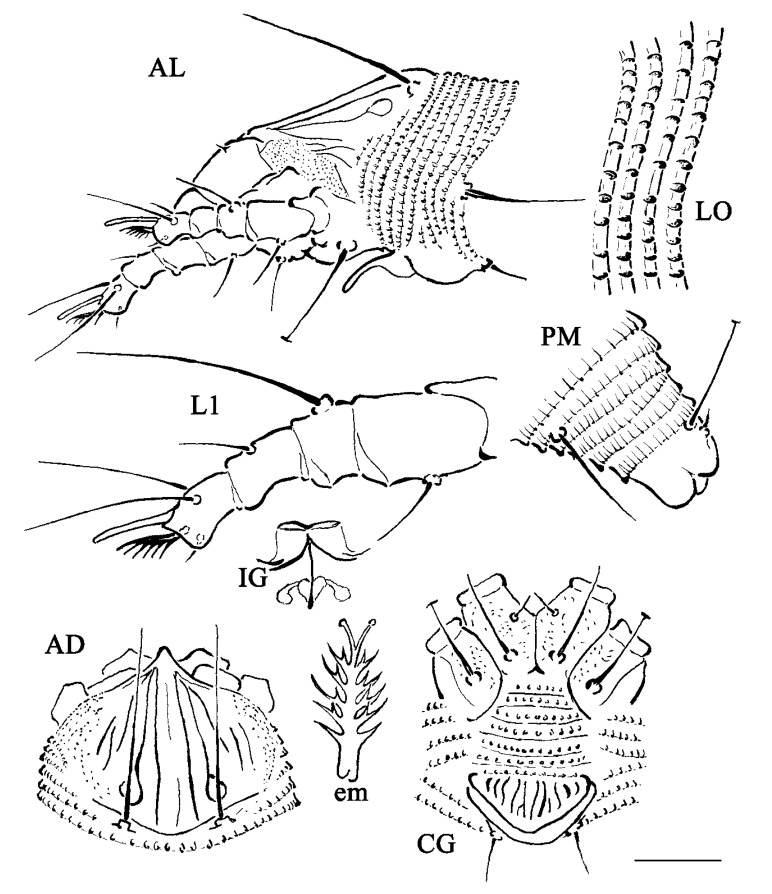
Line drawings of *Eriophyes pouteriae* sp. nov.: AD. Prodorsal shield; AL. Lateral view of anterior body region; CG. Female coxigenital region; em. Empodium; IG. Internal female genitalia; LO. Lateral view of annuli; L1. Leg I; PM. Lateral view of posterior opisthosoma. Scale bar: 10 μm for AD, AL, CG, IG, PM; 5 μm for LO, L1; 2.5 μm for em.

**Table 1:** Fragments amplified and primers utilized in PCR reactions and DNA sequencing for *Eriophyes pouteriae* sp. nov.

Region	Primer	Sequence for the Primer 5′–3′	Length (bps)
COI	LCO1490	GGTCAACAAATCATAAAGATATTGG	670 pbs
	HCO2198	TAAACTTCAGGGTGACCAAAAAATCA	
18S and 28S rRNA	fl230	TGAAACTTAAAGGAATTGACG	516 pbs
	D1D2 rev 04	GTTAGACTYCTTGGTCCGTG	
ITS	18S	AGAGGAAGTAAAAGTCGTAACAAG	900 pbs
	28SC	ATATGCTTAAATTCAGCGGG	

## Data Availability

The raw data concerning the mite description will be made available by the authors on request.
